# Semi-quantitative and quantitative dynamic contrast-enhanced (DCE) MRI parameters as prostate cancer imaging biomarkers for biologically targeted radiation therapy

**DOI:** 10.1186/s40644-022-00508-9

**Published:** 2022-12-19

**Authors:** Hayley M. Reynolds, Sirisha Tadimalla, Yu-Feng Wang, Maryam Montazerolghaem, Yu Sun, Scott Williams, Catherine Mitchell, Mary E. Finnegan, Declan G. Murphy, Annette Haworth

**Affiliations:** 1 Auckland Bioengineering Institute, The University of Auckland, Auckland, New Zealand; 2 School of Physics, The University of Sydney, Sydney, NSW Australia; 3 Division of Radiation Oncology, Peter MacCallum Cancer Centre, Melbourne, VIC Australia; 4 Sir Peter MacCallum Department of Oncology, The University of Melbourne, Melbourne, VIC Australia; 5 Department of Pathology, Peter MacCallum Cancer Centre, Melbourne, VIC Australia; 6 Department of Imaging, Imperial College Healthcare NHS Trust, London, UK; 7 Department of Bioengineering, Imperial College London, London, UK; 8 Division of Cancer Surgery, Peter MacCallum Cancer Centre, Melbourne, VIC Australia

**Keywords:** Dynamic contrast enhanced MRI, Imaging biomarker, Prostate cancer, Radiation therapy

## Abstract

**Background:**

Biologically targeted radiation therapy treatment planning requires voxel-wise characterisation of tumours. Dynamic contrast enhanced (DCE) DCE MRI has shown promise in defining voxel-level biological characteristics. In this study we consider the relative value of qualitative, semi-quantitative and quantitative assessment of DCE MRI compared with diffusion weighted imaging (DWI) and T2-weighted (T2w) imaging to detect prostate cancer at the voxel level.

**Methods:**

Seventy prostate cancer patients had multiparametric MRI prior to radical prostatectomy, including T2w, DWI and DCE MRI. Apparent Diffusion Coefficient (ADC) maps were computed from DWI, and semi-quantitative and quantitative parameters computed from DCE MRI. Tumour location and grade were validated with co-registered whole mount histology. Kolmogorov–Smirnov tests were applied to determine whether MRI parameters in tumour and benign voxels were significantly different. Cohen’s d was computed to quantify the most promising biomarkers. The Parker and Weinmann Arterial Input Functions (AIF) were compared for their ability to best discriminate between tumour and benign tissue. Classifier models were used to determine whether DCE MRI parameters improved tumour detection versus ADC and T2w alone.

**Results:**

All MRI parameters had significantly different data distributions in tumour and benign voxels. For low grade tumours, semi-quantitative DCE MRI parameter time-to-peak (TTP) was the most discriminating and outperformed ADC. For high grade tumours, ADC was the most discriminating followed by DCE MRI parameters Ktrans, the initial rate of enhancement (IRE), then TTP. Quantitative parameters utilising the Parker AIF better distinguished tumour and benign voxel values than the Weinmann AIF. Classifier models including DCE parameters versus T2w and ADC alone, gave detection accuracies of 78% versus 58% for low grade tumours and 85% versus 72% for high grade tumours.

**Conclusions:**

Incorporating DCE MRI parameters with DWI and T2w gives improved accuracy for tumour detection at a voxel level. DCE MRI parameters should be used to spatially characterise tumour biology for biologically targeted radiation therapy treatment planning.

## Background

Dynamic Contrast Enhanced (DCE) MRI is an effective tool to assess tissue perfusion and permeability, which are increased in prostate cancer (PCa). It is used alongside T2-weighted (T2w) and Diffusion Weighted Imaging (DWI) within a multiparametric MRI (mpMRI) protocol to detect and diagnose clinically significant PCa via the Prostate Imaging and Reporting System (PI-RADS) [1]. Recent updates to PI-RADS have reduced the importance of DCE MRI, however, and some now suggest a biparametric MRI (bpMRI) approach may be sufficient for tumour detection by obtaining T2w and DWI alone. This has been motivated by a desire to reduce time and costs, alongside reducing the risks associated with contrast agent injection. Studies have been performed to compare bpMRI against mpMRI, with some concluding they are comparable [2–5], whilst others have concluded that mpMRI is better because DCE MRI is beneficial for assessing higher PI-RADS lesions [4, 6].

DCE MRI offers more than detecting clinically significant cancers, however, as it provides underlying biological information about tumours which cannot be replaced by other imaging sequences [7]. For radiation therapy applications, studies have shown that DCE MRI is valuable for delineating tumours and can be used as a tool to inform dose painting approaches [8, 9]. Further, information from DCE MRI is complimentary to T2w and DWI, where T2w imaging is generally used for anatomical definition of treatment volumes and organs at risk, while DWI has been linked to cellularity and improves the reliability in differentiating between benign and clinically significant disease.

The biological characteristics of a patient’s tumour derived from DCE MRI can be used alongside T2w and DWI during the optimisation process for biologically targeted radiation therapy treatment planning [10]. Biologically targeted radiation therapy is an advanced form of dose painting whereby individual voxels are characterised by specific biological characteristics and termed “dose painting by numbers” [11]. Biological characteristics include those which are known to impact the biological effect of the dose delivered including radiosensitivity, the rate of tumour proliferation, and the presence of hypoxia [12], and can be derived from quantitative imaging in combination with mathematical modelling approaches [10]. Further work is required, however, to optimise the imaging parameters used to derive tumour characteristics for biologically targeted radiation therapy treatment planning, including those from DCE MRI.

Depending on the time resolution of image acquisition and the processing software used, DCE MRI can be analysed in different ways with increasing levels of complexity. The simplest analysis is performed through qualitative assessment of the T1-weighted images by manual inspection. More complex assessments are made by fitting a curve to the dynamic signal in each voxel and extracting parameters from the curve, termed ‘semi-quantitative’ parameters. Further to this, ‘quantitative’ kinetic parameters are computed by pharmacokinetic modelling of the dynamic signal or dynamic contrast agent concentrations derived from the dynamic signal [13]. A variety of methods and software packages exist to compute these parameters, with many inputs required such as the arterial input function (AIF) for pharmacokinetic modelling approaches [14]. Several prior studies have compared the ability of DCE MRI parameters to identify PCa [15, 16, 17, 18, 19] with many focusing on their ability to differentiate lesions based on Gleason Scores [19, 20, 21, 22]. However, each of these studies have been limited to assessing regions-of-interest (ROIs) only, many analysed semi-quantitative parameters alone or quantitative parameters alone, and most lacked co-registration with whole mount histopathology for ground truth validation.

In this study we investigated the ability of semi-quantitative and quantitative DCE MRI parameters to detect PCa in comparison with DWI and T2w imaging, using co-registered whole mount histopathology for validation. Analysis was carried out at a voxel-wise level, in contrast to prior studies in this field which used an ROI-based approach. The goal was to identify DCE MRI parameters which could provide complimentary information at a voxel-wise level, alongside T2w and DWI, to characterise the spatial distributions of tumour biology. Such voxel-wise spatial distribution maps of tumour biology could be used to plan biologically targeted radiation therapy, to personalise treatment whilst maximising treatment efficacy [10, 12].

## Methods

### Patients

Seventy PCa patients scheduled for radical prostatectomy between April 2013 and September 2018 at the Peter MacCallum Cancer Centre, Melbourne, were recruited to this Human Research Ethics Committee approved study (HREC/15/PMCC125), with all patients providing written informed consent. Imaging data from 61 of these patients were suitable for this study, while the remaining nine either did not have complete data for co-registration with histology or motion artefacts on DCE MRI which rendered the images unreliable. Table 1 details clinical and pathological information across the 61-patient cohort. The Gleason Score and Grade Group of the dominant intraprostatic lesion are given, although all tumour foci were used in the analysis.

**Table 1:** Patient clinical and pathological details. Gleason Score, Grade Group and PIRADS v2 (1) are reported for the dominant intraprostatic lesion. PSA = prostate specific antigen

**Variable (*n* = 61)**	**Mean**	**Range**
Age	62	(45, 74)
PSA (ng/mL)	9.0	(2.2, 42)
	**n (%)**	
Gleason Score / Grade Group		
- 3 + 3 / 1	4 (6.6)	
- 3 + 4 / 2	31 (50.8)	
- 4 + 3 / 3	20 (32.8)	
- 4 + 5 / 5	3 (4.9)	
- 5 + 4 / 5	3 (4.9)	
Extraprostatic extension	27 (44.3)	
Pathological T Stage		
- pT2	30 (49.2)	
- pT3a	23 (37.7)	
- pT3b	8 (13.1)	
PIRADs v2		
- 1	1 (1.6)	
- 2	7 (11.5)	
- 3	10 (16.4)	
- 4	10 (16.4)	
- 5	31 (50.8)	
- indeterminate	2 (3.3)	

### MRI data acquisition

Multiparametric MRI was obtained at 3T from each patient before prostatectomy, with the first 32 patients scanned on a Siemens MAGNETOM Trio (Siemens Healthineers, Erlangen, Germany) and the remaining 25 patients using a Siemens MAGNETOM Skyra. A surface body coil was used, without an endorectal coil to reduce the chance of deformation to the prostate during scanning. Patients without contraindications were given Buscopan to reduce rectal peristaltic motion. The imaging protocol was based on the European Society of Urogenital Radiology (ESUR) guidelines [23], and included T2w, DWI, and DCE imaging.

T2w imaging was obtained using a 2D turbo spin echo sequence using acquisition matrix = 320 × 320, FOV = 160 mm × 160 mm, slice thickness = 3 mm, TE = 89 – 96 ms, TR = 3500 – 4830 ms. DWI images were obtained using a 2D spin echo sequence with echo planar readout, with b-values = 50, 400, 800 and 1200 s/mm^2^, acquisition matrix = 250 × 250, FOV 250 mm × 250 mm, slice thickness = 4 mm. Apparent Diffusion Coefficient (ADC) maps were computed from DWI using inline software. Pre-contrast 3D T1-weighted images with variable flip angles (5º, 10º, 15º, 20º, 30º) were acquired. DCE-MRI was performed using a 3D spoiled gradient echo with a time-resolved view sharing sequence for high temporal resolution imaging (TWIST, Siemens Healthineers, Erlangen, Germany). Each patient received a 10ml bolus injection of contrast agent Dotarem (gadoterate meglumine, Guerbet, USA), followed by a saline flush. For the first eight patients, DCE-MRI data was collected using acquisition matrix = 256 × 256, FOV = 200 × 200 mm, flip angle = 20º with 16 transverse partitions at 4 mm section thickness and repeated 60 times at 7.2 s intervals except one patient with 20 transverse partitions at 3.5 mm section thickness, repeated 90 times at 5.3 s intervals to improve coverage of the small prostate. For the remaining patients, the DCE temporal resolution was increased to 120 times at 3.6 s intervals using a reduced spatial resolution with acquisition matrix = 128 × 128, FOV = 200 mm × 200 mm.

**Fig. 1:**
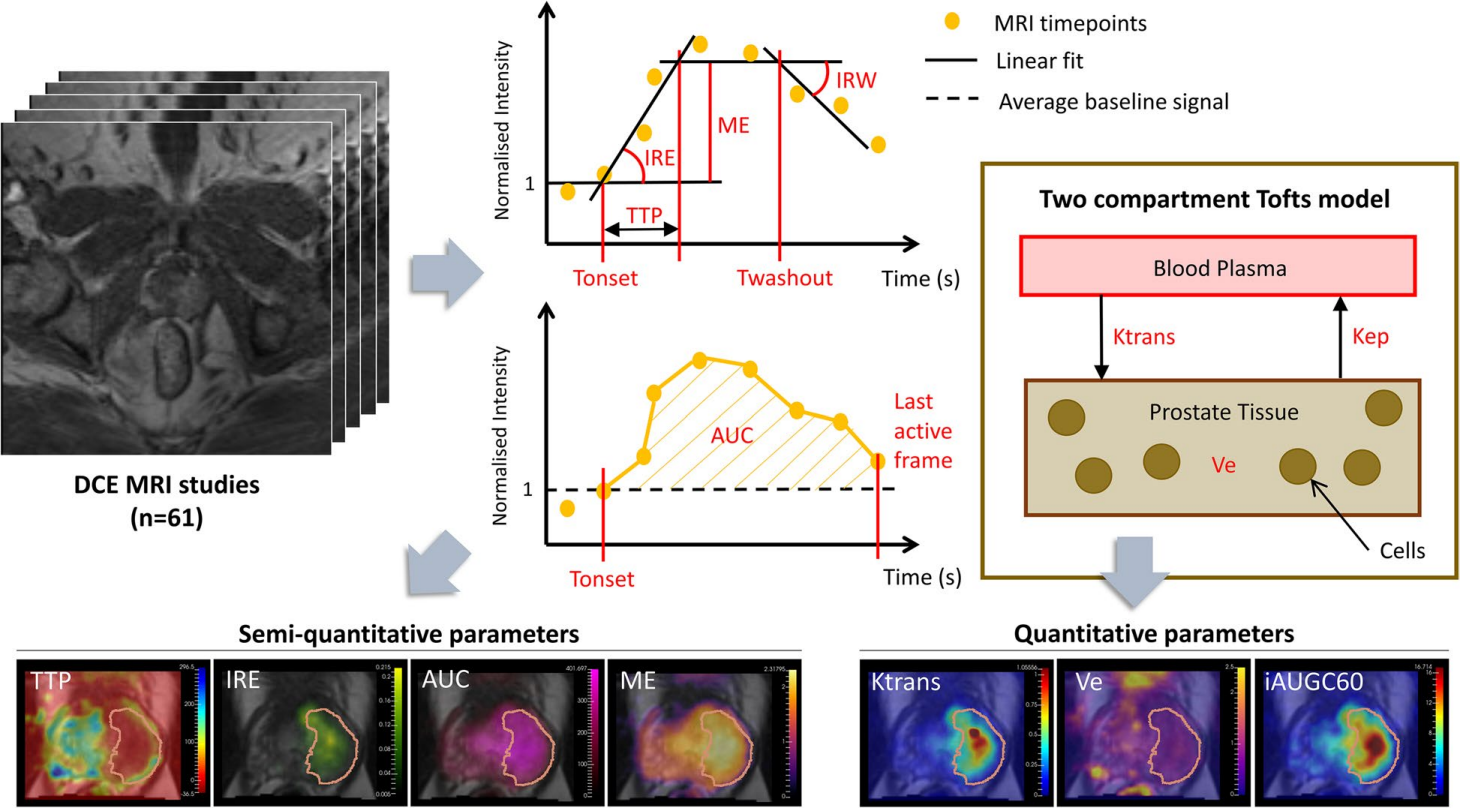
Semi-quantitative parameters were computed from the DCE MRI time series of each patient by fitting a piece-wise linear function to the signal intensity of each voxel, and AUC extracted from the normalised signal intensity curve. Quantitative maps Ktrans and Ve were computed by fitting the Tofts pharmacokinetic model to the DCE MRI data and the iAUGC60 parameter from the area under the Gadolinium contrast agent concentration curve

Semi-quantitative parametric maps were computed from the DCE MRI data using Dynamika software (Image Analysis Group, London, UK) [24] by fitting a continuous piecewise linear function to the signal intensity curve of each voxel (see Fig. 1). Parameters extracted from this linear function included the initial rate of enhancement (IRE) which is the slope of the enhancement phase, the time to peak enhancement (TTP) which is the difference between the start of enhancement and the plateau phase, the maximum enhancement (ME), the time of contrast agent onset (T_onset_), the time of contrast agent washout (T_washout_), the initial rate of washout (IRW) which is the slope of the washout phase and the area under the curve (AUC) which was the area between the baseline intensity and the normalised intensity curve.

DCE signals were converted to concentrations of the contrast agent. Relaxivity of the contrast agent was 3.5 Lmmol^−1^ s^−1^ [25], and the T1 value for the arterial blood relaxation time was fixed at 1664 ms [26]. The motion in some patients’ pre contrast variable flip angle scans was found to impact the accuracy of tissue T1 calculation and therefore a fixed T1 value of 1597 ms [27] in the prostate for all patients was used for consistency. The initial area under the Gadolinium contrast agent concentration curve for the first 60 s post-injection (iAUGC60) was computed. The arterial input function was defined using two population-based AIFs: the Weinmann model [28] and the Parker model [29]. Pharmacokinetic parameters Ktrans (the volume transfer constant between blood plasma and extra-vascular extra-cellular space) and Ve (volume of extra-vascular extra-cellular space) of the Tofts model were obtained as shown in Fig. 1, using Dynamika software (Image Analysis Group, London, UK) [24, 30].

### Co-registration with histology

In vivo mpMRI was co-registered with high resolution Haematoxylin and Eosin (H&E) stained histology using an established framework previously reported [31]. In brief, the H&E-stained histology slides were annotated by an experienced urological pathologist (CM) for tumour location and Gleason Score and digitised using an Epson Perfection V700 scanner (Epson, Suwa, Japan). Co-registration of in vivo mpMRI with histology included an intermediate step utilising ex vivo MRI of the prostate specimen and a combination of rigid and deformable registration to account for tissue shrinkage and deformation of the prostate caused by specimen removal and histology processing. All co-registered in vivo mpMRI maps and histology data were resampled into isotropic voxels to match the 3D T2w MRI used in the registration process which had resolution of 0.8 × 0.8 × 0.8 mm.

**Fig. 2:**
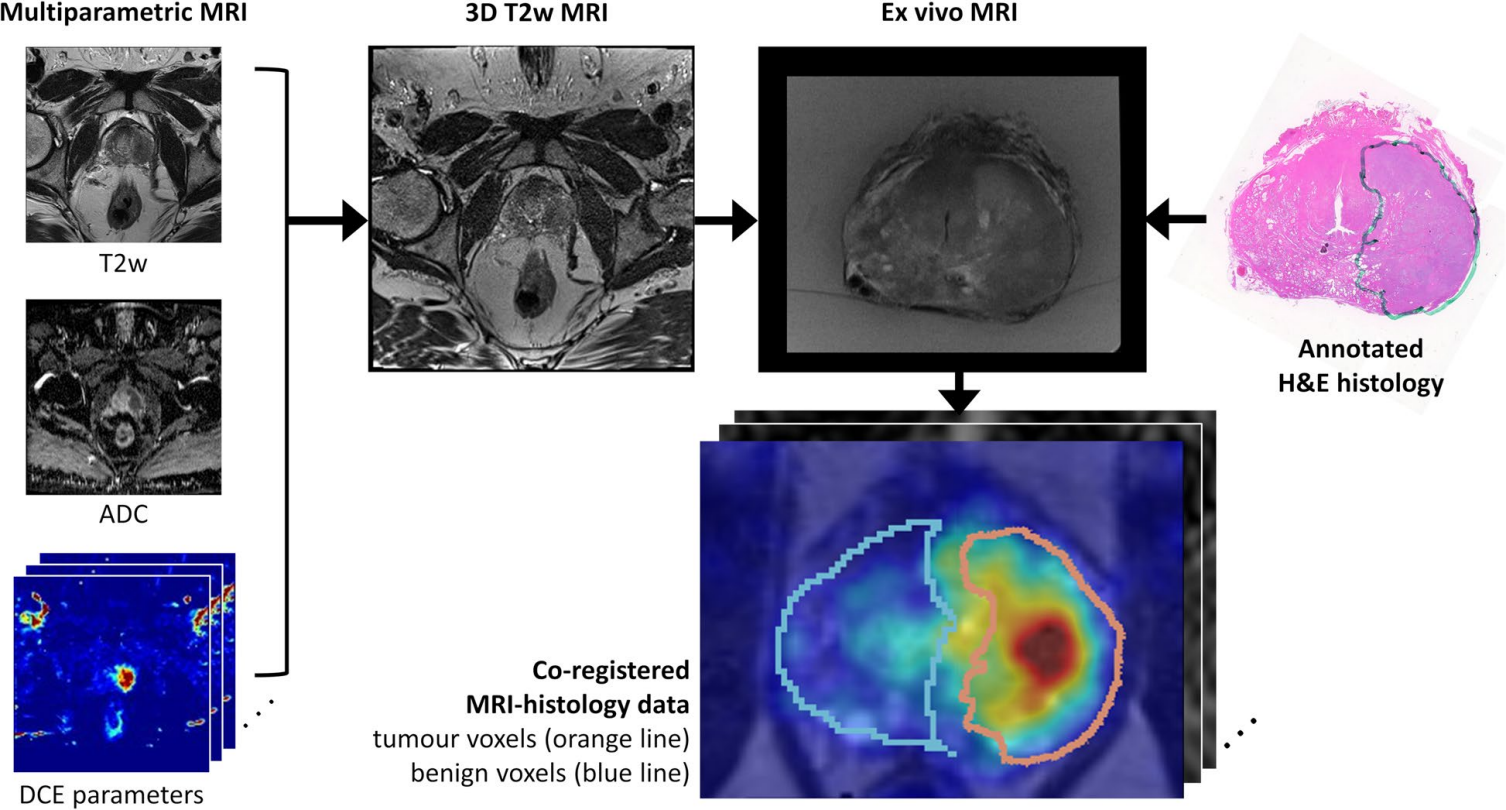
Multiparametric MRI including T2w, ADC and DCE MRI semi-quantitative and quantitative maps were rigidly registered with in vivo 3D T2w MRI, then deformably registered with ex vivo MRI and histology data. Tumour voxels (orange outline) were derived from pathologist’s annotations on co-registered histology, and benign voxels (blue outline) were defined as all prostate voxels 3.3 mm beyond this tumour boundary

Mask images created from the annotated histology slides were used to define tumour voxels in the co-registered mpMRI. To define benign voxels, the tumour annotation masks were dilated by 3.3 mm to account for registration uncertainty calculated in a prior study [31] and all voxels outside this boundary were considered benign. Hence there was a small proportion of voxels around the tumour annotation mask which were not categorised as tumour or benign and therefore not analysed due to the registration uncertainty (see Fig. 2).

### Statistical analysis

The statistical analysis carried out was based on the following questions: (1) does each MRI parameter within tumour tissue have a significantly different data distribution compared with benign tissue?; (2) which DCE MRI parameter is the best imaging biomarker for identifying tumours on MRI?; (3) which population-based AIF function between the Parker and Weinmann models used to compute pharmacokinetic parameters Ktrans and Ve best distinguishes between tumour and benign voxel values?; and (4) what improvement in a computer aided detection (CAD) system will DCE MRI parameters provide when combined with T2w and ADC? Each of these questions were addressed at a voxel-wise level by assessing all tumour voxels together, then separately according to those with low-grade disease defined as tumour voxels with Gleason Score <  = 3 + 4 / Grade Group <  = 2 and tumour voxels with high-grade disease defined as Gleason Score >  = 4 + 3 / Grade Group >  = 3. For each analysis DCE MRI parameters Tonset, Twashout and IRW were excluded. This was due to the Tonset parameter being inconsistent across the dataset and challenging to reproduce, while Twashout and IRW contained many zero value pixels as the contrast agent had not washed out from the entire prostate during the imaging timeframe.

To first assess the difference in MRI parameter values between tumour and benign voxels, two-sample Kolmogorov Smirnov tests were used. To address the second and third questions, Cohen’s d was computed, which is a type of effect size measure to compare the difference between the mean of two groups [32]. A higher Cohen’s d value indicates a larger difference between the two groups, where a common interpretation is a negligible effect size is < 0.2, a small effect size is 0.2 to 0.5, medium effect size is 0.5 to 0.8 and large effect size is > 0.8. In this analysis the means considered were the tumour voxel values and benign voxel values for each MRI parameter.

To answer the last question to determine how DCE MRI parameters improve tumour detection in a CAD system when used along with ADC and T2w MRI, Logistic Regression (LR) and Random Forest (RF) classifier models were used. Only 57 of the original 61 patients were used for this step, as four patients had artefacts on ADC maps caused by rectal gas or motion during scanning and therefore were not suitable. Data was first normalised, and models trained using 80% voxels and the remaining 20% voxels were used as test data. To address class imbalance due to substantially more benign voxels (1,059,147) than tumour voxels (228,725), the benign voxels were down sampled using random sampling to match the number of tumour voxels more closely.

Multiple LR and RF models were fit to the data to assess the importance of DCE MRI. Firstly, by fitting models using T2w and ADC alone, then adding Ktrans (the most reported DCE MRI parameter), and then three additional models with systematic addition of DCE MRI parameters to determine which would give the best tumour prediction performance. These models utilised semi-quantitative DCE parameters alone, quantitative DCE parameters alone, then both semi-quantitative and quantitative DCE parameters, each excluding highly correlated parameters identified by computing Pearson correlation coefficients. Performance metrics sensitivity, specificity and accuracy were computed for all models, and feature importance assessed.

## Results

As shown in Table 1, there were 35 patients (57%) with low grade index lesions (four with Gleason Score 3 + 3 / Grade Group 1 index lesions and 31 with Gleason Score 3 + 4 / Grade Group 2 index lesions), and the remaining 26 patients (43%) had high grade index lesions (Gleason Score >  = 4 + 3 / Grade Group >  = 3), all of whom had PIRADS v2 scores on mpMRI of 3 and above. Over 80% of patients had a PIRADS v2 lesion of 3 or above, with two patients classified as indeterminate due to artefacts on ADC maps.

### Distribution of tumour and benign voxel values

Kolmogorov–Smirnov tests showed that data distributions between tumour and benign voxel values for all MRI parameters, both for all tumours and when tumours were separated into high grade and low grade, were significantly different to benign voxel values and could be considered to come from different data distributions.

**Fig. 3:**
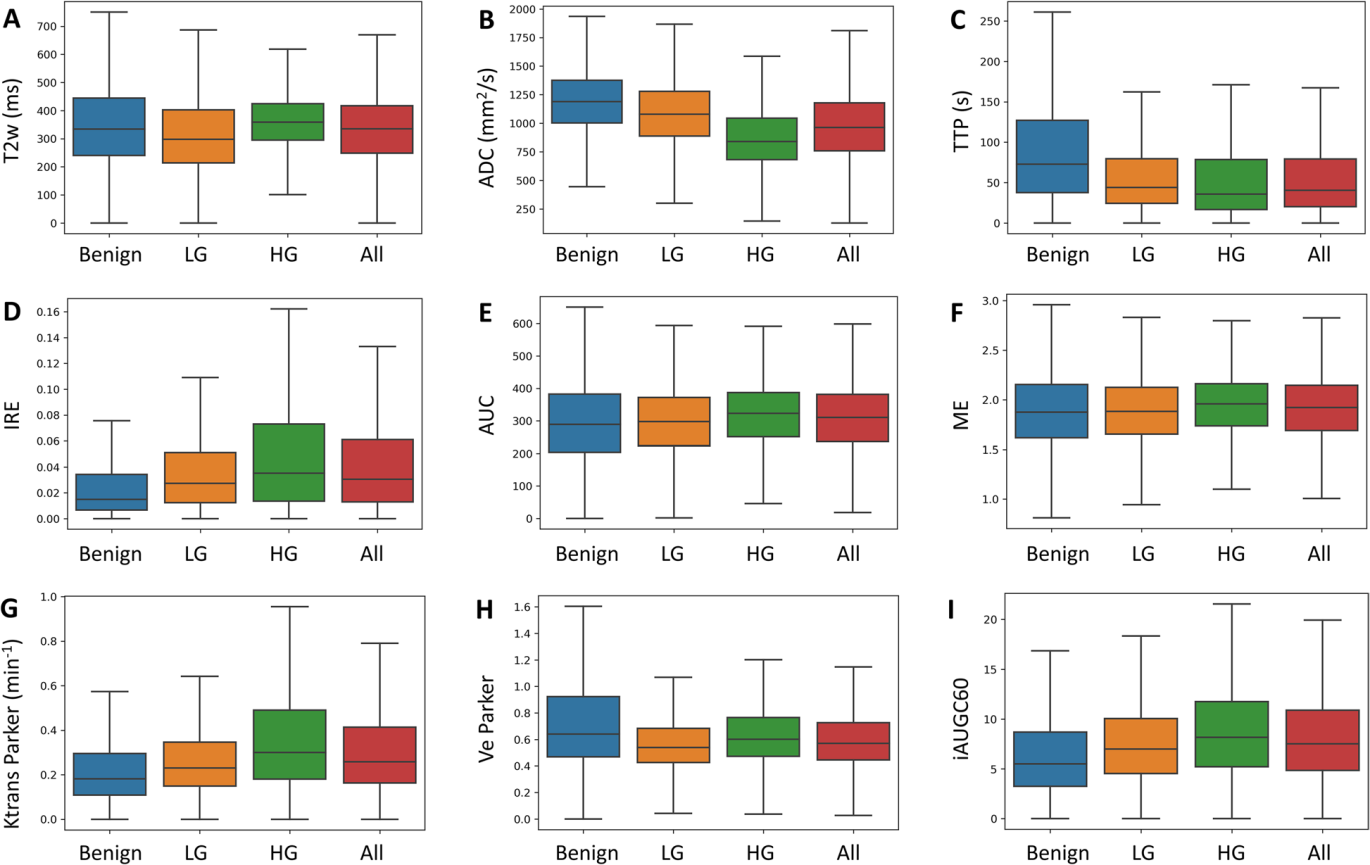
Box and whisker plots (excluding outliers) showing the distribution of benign, low grade (LG), high grade (HG) and all tumour voxel values from (**a**) T2w, (**b**) ADC, (**c**) TTP, (**d**) IRE, (**e**) AUC, (**f**) ME, (**g**) Ktrans Parker, (**h**) Ve Parker and (**i**) iAUGC60

Figure 3 shows box and whisker plots for selected parameters, comparing the distribution of benign voxel values with low grade and high grade tumour voxel values. The mean DCE MRI parameters Ktrans, IRE, iAUGC60, AUC and ME were higher in tumour than benign tissue, while the mean ADC and DCE MRI parameter TTP were lower in tumours when compared with benign tissue.

### Potential imaging biomarkers from DCE MRI

Table 2 details the Cohen’s d values for each MRI parameter, ordered from maximum to minimum based on the absolute value. Cohen’s d values show that ADC was the most discriminating MRI parameter for tumours overall, with the largest effect size of 0.959 for high grade tumours and medium effect size of 0.644 for all tumours combined. However, for low grade tumours, the DCE MRI parameter TTP was shown to be more discriminating than ADC with a Cohen’s d value of 0.478 versus 0.323.

**Table 2:** Absolute Cohen’s d values for each MRI parameter comparing benign voxels versus low grade tumour, high grade tumour and all tumour voxels. MRI parameters ordered by highest to lowest absolute Cohen’s d value based on all tumours. Cohen’s d values < 0.2 represent a negligible effect size, while values above 0.2, 0.5 and 0.8 represent small, medium and large effect sizes respectively

**MRI Parameter**	**Low Grade Tumours**	**High Grade Tumours**	**All Tumours**
ADC	0.323	0.959	0.644
TTP	0.478	0.548	0.524
Ktrans Parker	0.264	0.759	0.500
Ktrans Weinmann	0.217	0.701	0.453
IRE	0.255	0.591	0.416
iAUGC60	0.252	0.513	0.384
Ve Parker	0.315	0.241	0.286
Ve Weinmann	0.267	0.286	0.277
T2w	0.221	0.045	0.090
AUC	0.030	0.084	0.028
ME	0.022	0.053	0.016

When assessing the DCE MRI parameters, four had a large effect size (Cohen’s d over 0.8) for high grade disease: Ktrans, IRE, TTP and iAUGC60, with TTP and Ktrans also having Cohen’s d over 0.8 for all tumours combined. For low grade tumours, there were small to negligible effect size values given for all DCE MRI parameters. All tumours combined, as well as both high grade and low grade tumours showed the following order of discrimination: ADC, Ktrans, IRE, iAUGC60, T2w. Both AUC and ME parameters had negligible effect size (Cohen’s d < 0.2).

### Parker versus Weinmann Population-based AIF function

Utilising the Parker AIF to compute Ktrans and Ve was more discriminating for all tumours than the Weinmann AIF. For all tumours Ktrans Parker had a Cohen’s d value of 0.500 for the Parker AIF and 0.453 for the Weinmann AIF, while Cohen’s d for Ve Parker was higher than Ve Weinmann for all tumours (0.286 versus 0.277) and low grade tumours (0.315 versus 0.267). Hence, for the subsequent CAD models, Ktrans and Ve parameters computed with the Parker AIF have been used.

### CAD Models with and without DCE MRI Parameters

**Fig. 4:**
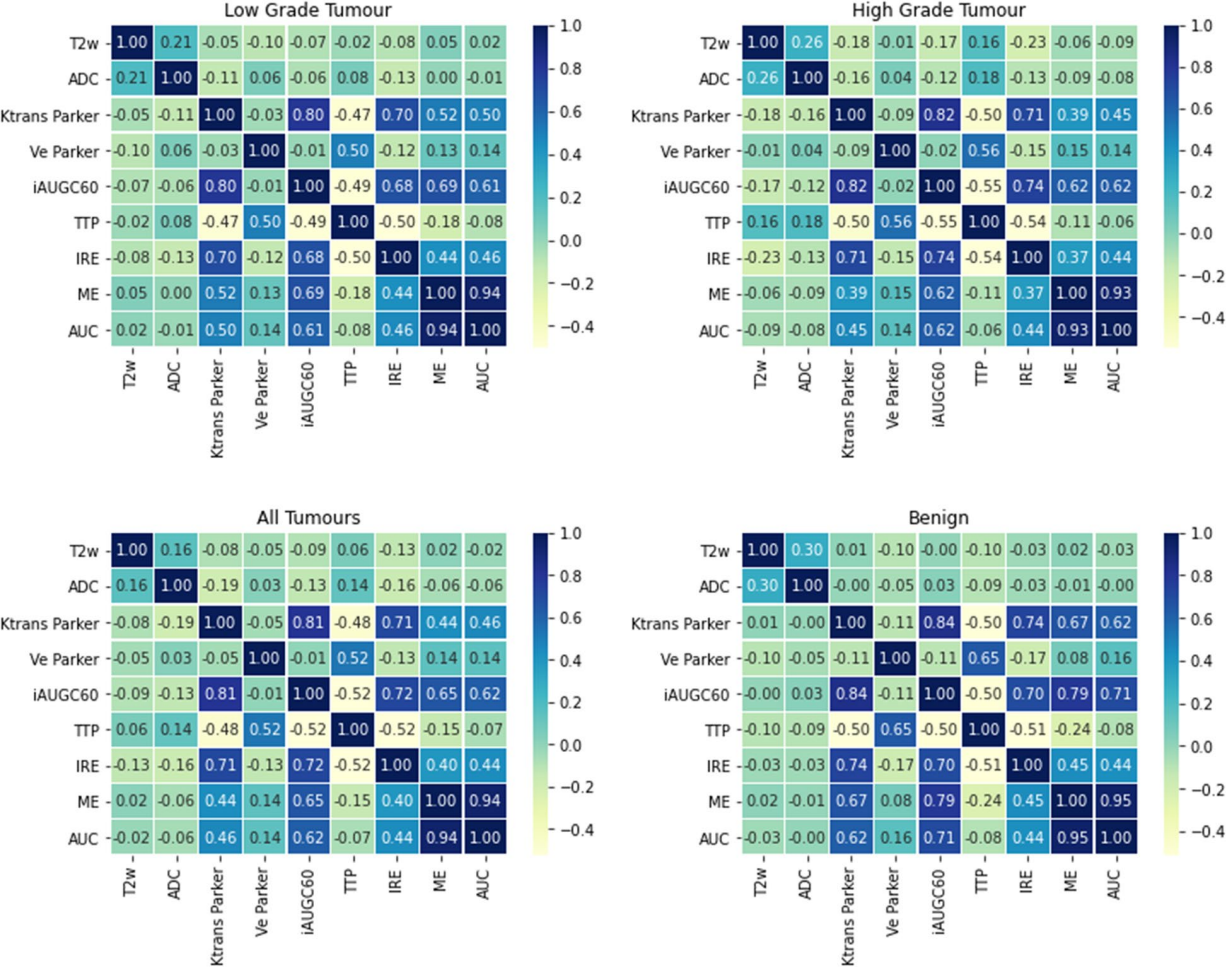
Heatmaps showing the degree of correlation between each MRI parameter in low grade (top left) and high grade tumours (top right), all tumour voxels (bottom left) and benign voxels (bottom right). Ktrans and Ve were computed using the Parker AIF

Correlation coefficients for each MRI parameter are shown in Fig. 4, for low grade, high grade, all tumours and benign voxel values. The T2w and ADC values showed very low correlations with all other MRI parameters, with the largest correlation between T2w and ADC in benign voxels of 0.30. DCE MRI parameter Ktrans highly correlated with both iAUGC60 (range 0.80 to 0.84) and IRE (range 0.70 to 0.74). In addition, iAUGC60 was highly correlated with IRE (range 0.68 to 0.74), and ME (range from 0.62 to 0.79). The AUC parameter showed the highest correlation of all parameters with ME (ranging between 0.93 to 0.95). Hence, the CAD model utilising quantitative parameters with no high correlations excluded iAUGC60 which left Ktrans and Ve. The CAD model with semi-quantitative DCE MRI parameters excluded ME which left TTP, IRE and AUC. Lastly the CAD model which combined semi-quantitative and quantitative parameters excluded those with a correlation coefficient over 0.65 which left Ktrans, Ve, TTP and AUC.

Table 3 details the performance metrics for each of the CAD classifier models. Overall, the RF classifier performed better than the LR classifier when DCE MRI parameters were included, higher sensitivity and accuracy values (the one exception is the low grade tumour model with T2w, ADC and Ktrans, which gave equal sensitivity between the two classifiers of 63%). In contrast the LR classifier performed better overall than the RF classifier when just T2w and ADC parameters alone were used. Furthermore, the LR models appeared to reach a plateau in accuracy when more DCE MRI parameters were added, suggesting the models were overfitting in contrast to the RF models which continued to increase in accuracy with the addition of more DCE MRI parameters.

**Table 3:** Performance metrics from each of the Logistic Regression and Random Forest classifier models, where low grade tumours have Gleason Score <  = 3 + 4 / Grade Group <  = 2 and high grade tumours have Gleason Score >  = 4 + 3 / Grade Group >  = 3. The best performing metrics when comparing the two classifiers are in bold. Ktrans and Ve were computed using the Parker AIF

**MRI Parameters**	**Logistic Regression Models**			**Random Forest Models**		
**Low Grade Tumours**	Sensitivity	Specificity	Accuracy (%)	Sensitivity	Specificity	Accuracy (%)
T2w + ADC	0.21	**0.89**	**60**	**0.40**	0.70	58
T2w + ADC + Ktrans	0.25	**0.88**	**63**	**0.44**	0.77	**63**
T2w + ADC + Ktrans + Ve	0.38	**0.84**	65	**0.57**	0.81	**71**
T2w + ADC + TTP + IRE + AUC	0.48	0.79	66	**0.63**	**0.83**	**74**
T2w + ADC + Ktrans + Ve + TTP + AUC	0.47	0.79	66	**0.68**	**0.86**	**78**
**High Grade Tumours**	Sensitivity	Specificity	Accuracy (%)	Sensitivity	Specificity	Accuracy (%)
T2w + ADC	**0.65**	**0.81**	**74**	0.63	0.79	72
T2w + ADC + Ktrans	0.64	0.82	75	**0.68**	**0.84**	**77**
T2w + ADC + Ktrans + Ve	0.65	0.83	75	**0.72**	**0.86**	**80**
T2w + ADC + TTP + IRE + AUC	0.65	0.82	75	**0.76**	**0.87**	**82**
T2w + ADC + Ktrans + Ve + TTP + AUC	0.65	0.83	76	**0.79**	**0.89**	**85**
**All Tumours**	Sensitivity	Specificity	Accuracy (%)	Sensitivity	Specificity	Accuracy (%)
T2w + ADC	**0.56**	**0.74**	**66**	0.54	0.69	62
T2w + ADC + Ktrans	0.56	0.76	66	**0.57**	**0.77**	**68**
T2w + ADC + Ktrans + Ve	0.60	0.75	68	**0.64**	**0.79**	**72**
T2w + ADC + TTP + IRE + AUC	0.62	0.74	69	**0.68**	**0.81**	**75**
T2w + ADC + Ktrans + Ve + TTP + AUC	0.61	0.75	68	**0.72**	**0.84**	**80**

When assessing the RF models, adding T2w and ADC maps with semi-quantitative DCE MRI parameters alone (TTP + IRE + AUC) gave better detection performance overall when compared with adding quantitative DCE MRI parameters alone (Ktrans + Ve). The detection accuracy for high grade tumours was 82% for semi-quantitative parameters and 80% for quantitative parameters, whereas accuracy for low grade tumours was 74% versus 71% respectively. Similarly, sensitivity for high grade tumours was 0.76 for semi-quantitative parameters versus 0.72 for quantitative parameters, while low grade tumours achieved 0.63 versus 0.57 for each respectively. Specificity was higher than sensitivity for all CAD models. The RF models which used both semi-quantitative and quantitative DCE MRI parameters (Ktrans + Ve + TTP + AUC), achieved the highest performance metrics. High grade tumours were predicted with the highest accuracy of 85%, with sensitivity 0.79 and specificity of 0.89 whilst the low grade tumour model resulted in accuracy of 78% with sensitivity 0.68 and specificity of 0.86, and all tumours combined gave accuracy 80%, sensitivity 0.72 and specificity 0.84.

**Fig. 5:**
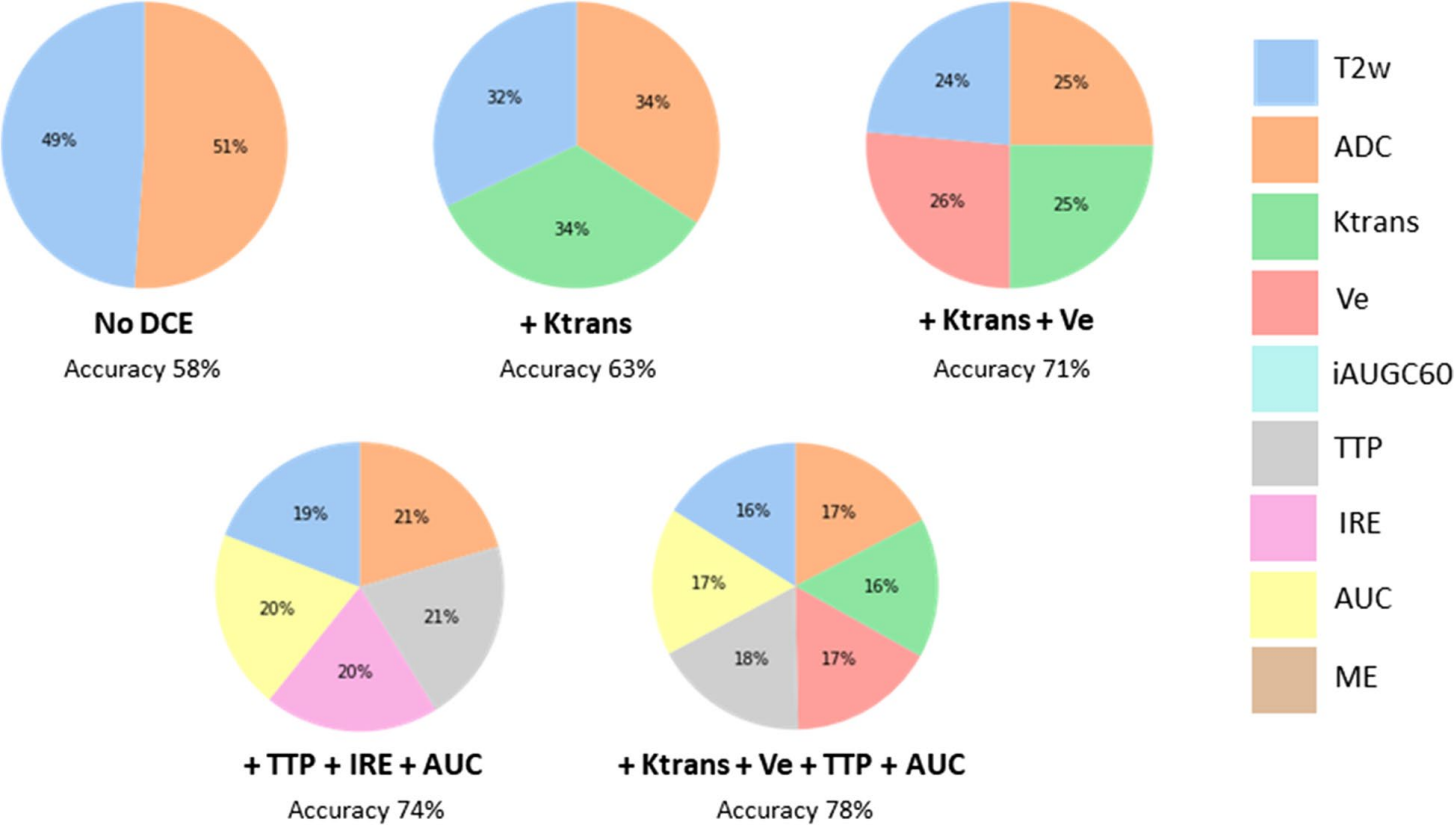
Pie charts showing the relative feature importance for MRI parameters in the RF models for low grade tumours. Ktrans and Ve computed using the Parker AIF

**Fig. 6:**
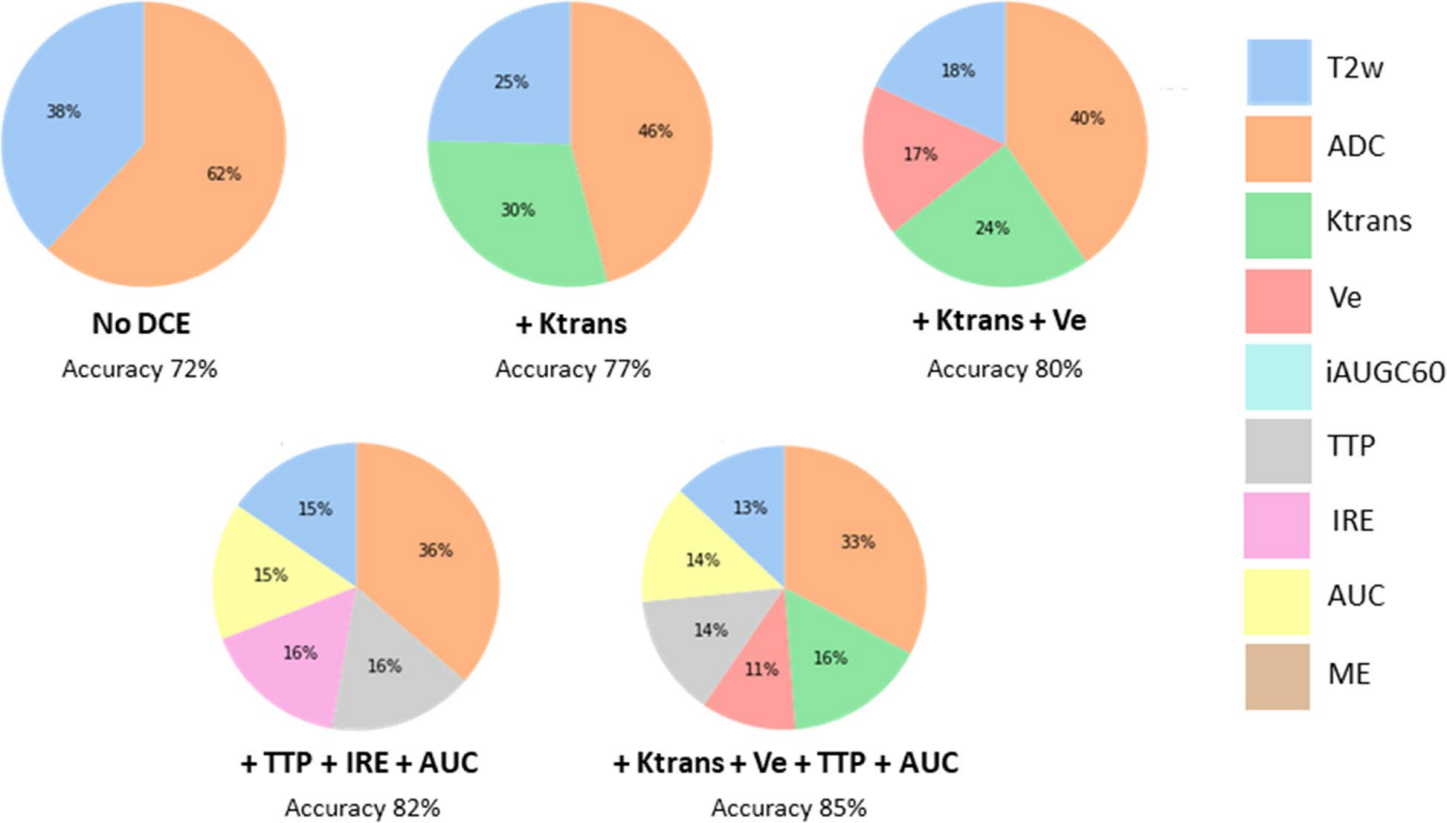
Pie charts showing the relative feature importance for MRI parameters in the RF models for high grade tumours. Ktrans and Ve computed using the Parker AIF

Figures 5 and 6 show pie charts with the relative feature importance ranking for MRI parameters in each low grade and high grade RF models. The ADC parameter was the most important individual feature overall, particularly predominant in the high grade tumour models ranging from 33 to 62% (Fig. 6). For the low grade tumour models, the feature importance for ADC ranged from 17 to 51% however when DCE parameters were included, Ktrans, Ve and TTP frequently matched or surpassed the ADC feature importance by 1%. Furthermore, there was no more than 3% difference between the feature importance of all parameters in the low grade models indicating a relatively even contribution from each MRI parameter.

For all high grade tumour models, when Ktrans was included it was the second most important parameter after ADC, ranging from 16 to 30%. The importance of T2w MRI in the RF models was consistently lower than for ADC and decreased with additional DCE MRI parameters. When quantitative or semi-quantitative DCE MRI parameters were included, they contributed between 34 – 67% in feature importance for the low grade models and between 30 – 54% for the high grade models.

## Discussion

The clinical utility of DCE MRI for detecting PCa has been the subject of recent debate, with its limited role for detecting clinically significant disease within the PIRADS scoring system. However, the PI-RADS Steering Committee recommend that mpMRI is preferrable over bpMRI when the priority is cancer detection [33] and that bpMRI could only be considered when image quality and radiology readings are of a high quality. DWI is the most prone to imaging artefacts and can have low signal-to-noise ratio [34], and therefore DCE remains important. Furthermore, DCE MRI contributes important biological information about tumour perfusion which cannot be replaced by other sequences. This information is particularly relevant for biologically targeted radiation therapy applications as it complements the information from T2w and DWI.

DCE MRI also has significant potential as a biomarker for monitoring response to RT [35, 36] as it can quantify changes to tissue perfusion and permeability. Spatial maps of tumour perfusion and permeability from DCE MRI can provide baseline features for monitoring response during treatment (for the purpose of biologically adaptive radiation therapy) or post treatment to detect recurrent disease. Whilst there has been a focus on imaging methods to detect only clinically significant PCa at diagnosis to avoid overtreatment of non-clinically significant disease, it could be argued that the focus should be to detect all cancers to differentiate between non-clinically significant and clinically significant disease. The latter could then receive treatment and the former be monitored for progression to a higher grade via active surveillance [37].

The aim in this study was to investigate the ability of DCE MRI parameters to detect PCa at a voxel-level in multiparametric MRI data for comparison with T2w and ADC maps. Key strengths of this study included the voxel-wise analysis using a highly controlled dataset, the accurate co-registration with ground truth histology data for all MRI parametric maps using an established framework [31], and the comprehensive set of semi-quantitative and quantitative DCE MRI maps. Another key strength was the separate assessment of low grade and high grade tumours, to determine whether the relative importance of MRI parameters would differ between the two groups and further inform the most appropriate approach for biologically targeted radiation therapy planning.

### Potential Imaging Biomarkers from DCE MRI

The most common DCE MRI imaging biomarkers used in clinical studies and trials to date are Ktrans and iAUGC [38]. Both quantitative parameters have been used to monitor the effect of oncology drugs including antiangiogenic or antivascular therapies, with many therapies showing a consistent reduction in Ktrans and/or iAUGC indicating a positive response [39]. Here, Ktrans was the most discriminating quantitative DCE MRI parameter whereas TTP was the most discriminating semi-quantitative DCE MRI parameter. For high grade tumours Ktrans was the most discriminating after ADC followed by semi-quantitative parameter IRE, then TTP, then iAUGC60. In contrast, TTP was the most discriminating parameter for low grade tumours, followed by ADC then Ktrans.

When comparing ADC with DCE MRI parameters, ADC was more discriminating for high grade tumours and for all tumours combined but not for low grade tumours where it was surpassed by TTP. The strong performance of TTP for tumour detection is consistent with several earlier studies. For example, Zhao et al. [17], investigated the correlation of six perfusion parameters with Ga-68 PSMA PET, and found that malignant lesions had significantly shorter TTP values than benign lesions and no other perfusion parameters (including Tonset, wash-in, washout, peak enhancement intensity and iAUC60) were significant. While in the study by Sung et al. [16], TTP was identified as one out of seven top DCE MRI parameters for detecting PCa.

In addition, several researchers have concluded that ‘wash-in’, equating to IRE used here, is a particularly significant semi-quantitative DCE MRI parameter for detecting tumours. This includes Isebeart et al. [18] who concluded that wash-in was the most accurate semi-quantitative parameter for discriminating between tumour and benign prostate tissue; Kim et al. [19] who assessed semi-quantitative DCE MRI parameters and concluded that wash-in was the most accurate for differentiating cancer foci for Gleason Score 8 and higher; and Sung et al. [16] who found that ‘wash-in-rate’ was a high performing DCE MRI parameter for detecting PCa. These results are consistent with the findings here where IRE was the most discriminating semi-quantitative parameter for high grade tumours. It was not, however, the most discriminating semi-quantitative parameter for low grade tumours or all tumours combined which was TTP in both cases.

### Parker versus Weinmann Population-based AIF function

Quantitative DCE MRI parameters are expected to be more robust than semi-quantitative parameters, as the raw signal intensity value is converted into contrast agent concentration. In theory this means quantitative DCE MRI parameter values should be more comparable between studies and centres because differences have been reduced, such as those caused by MRI scanner type, the data acquisition process, the contrast agent dose, and injection protocol used. Despite this, substantial variability can still exist in Ktrans and Ve values particularly due to the different models available and input parameters required to compute them such as the AIF [14]. Multi-centre studies have shown that AIF induced variations are larger for Ktrans than for Ve, however they are largely systematic with relatively little change to parametric map patterns within the prostate itself [40, 41].

In this study, obtaining a patient-specific AIF was problematic due to flow artefacts at the femoral arteries, so two different population-based AIFs were used. This included the Weinmann AIF [28] which has low-temporal-resolution and the Parker AIF, which has high-temporal resolution and shows a first pass and a recirculation peak, followed by a prolonged washout [29]. Results showed that Ktrans and Ve values computed using the Parker AIF was more discriminatory for tumour overall than the Weinmann AIF, however differences were small. The minor improvement in discriminatory power may be due to the Parker AIF having a more realistic time course for blood perfusion in the prostate due to its higher resolution and its mode of derivation. The Parker AIF was averaged from a larger cohort (67 DCE MRI studies from 23 patients) than the Weinmann AIF (derived using contrast agent measurements from 5 healthy volunteers).

Results can be compared with a study by Othman et al. [42] who computed Ktrans, Ve and Kep for 66 PCa patients using the Tofts model and three different AIFs, a Fritz-Hansen AIF [43] which was considered ‘fast’, the Parker AIF considered ‘intermediate’, and the Weinmann AIF considered ‘slow’. ROC analyses indicated all three AIFs had similar diagnostic accuracies however the Parker AIF gave the highest goodness of fit (chi^2^ value) of the Tofts model to the average signal from the lesion volumes. Another study by Azahaf et al. [14] found the Weinmann AIF was the best for distinguishing PCa from benign tissue when compared with a patient-specific AIF and the Fritz-Hansen AIF. However they did not include the Parker AIF in their analysis.

### CAD Models with and without DCE MRI Parameters

Numerous CAD models have been developed over the years to predict tumour location from mpMRI data, incorporating T2w, DWI and DCE MRI and often radiomics or deep learning techniques [44]. Here, the purpose was to utilise a series of CAD models to quantify the difference in tumour detection performance at a voxel-level with and without DCE MRI parameters. Results showed that when DCE MRI parameters were included the detection performance improved versus using T2w and ADC alone, with up to 20% improvement in accuracy for detecting high grade tumours and up to 13% improvement in accuracy for detecting low grade tumours.

The CAD model utilising semi-quantitative DCE parameters gave a 2–3% higher accuracy than when utilising quantitative DCE parameters alone. Despite the semi-quantitative DCE MRI parameters giving higher detection performance, this may not be reproducible in low resolution data when fewer post-contrast images are available. Therefore, utilising quantitative DCE MRI parameters for tumour detection is preferred, but if they are not available, then using semi-quantitative DCE MRI parameters for tumour detection is better than none.

When assessing the CAD model feature importance, the ADC parameter was the most dominant which is consistent with the Cohen’s d results where ADC was the most discriminating parameter overall. It should be recognised, however, that RF feature importance ranking cannot be directly compared because Cohen’s d evaluates each MRI parameter independently whereas the RF model involves an interaction of terms. The DWI sequence is the most prone to imaging artefacts and can have low signal-to-noise (SNR) ratio, so in these cases ADC would not be reliable as a predictive feature and DCE MRI parameters would be even more important.

Results can be compared with the study by Sung et al. [16] who developed a series of CAD models using an extensive set of 13 DCE MRI parameters, comparing their ability to detect PCa versus single DCE MRI parametric maps. Their study was strengthened by histopathology validation but was conducted using an ROI approach rather than the voxel-wise approach used here. Out of ten semi-quantitative parameters and three quantitative parameters, they concluded seven were accurate for PCa detection on their own (Kep, Kel, initial slope, slope50, wash-in rate, wash-out rate and TTP), but the CAD approach with all parameters was more accurate for tumour detection.

### Limitations and future work

There were study limitations, as data were only obtained from one centre and just one software package was used to compute DCE MRI parameters. The use of population-based AIFs and uniform T1 values rather than a T1 map was necessary due to artefacts in the data, however ideally these would have been determined for each individual patient. The highest b-value for DWI acquired was b = 1200, which was lower than the mandatory high b-value >  = 1400 which is now required for PIRADS version 2.1. Hence, voxel values from a high b-value diffusion-weighted images were not included in the analysis. Additionally, the voxels were not separated into peripheral and transitional zones, which would have enabled easier comparison with PIRADS v2.1 recommendations. Further, the uncertainty in the co-registered data was considered in the methodology by excluding voxels 3.3 mm around the annotated tumour based on our prior study results. However, this is uncertainty is an estimate, and sensitivity of the results to this value was beyond the scope of our study.

Future work will include analysing peripheral and transitional zones separately and computing radiomics and deep learning features to quantify tumour heterogeneity which may be more predictive of tumour location than the voxel-based parameters used here. In addition, an external validation study should be performed to determine whether the relative importance of DCE MRI parameters is consistent across different centres with differing imaging protocols. Lack of standardisation for DCE MRI is an ongoing challenge, particularly as differing imaging protocols and processing software can be used to derive DCE MRI parameters. The Quantitative Imaging Biomarkers Alliance (QIBA) [45, 46] provides guidelines for standardised acquisition and processing, and through the ongoing development of open access resources for perfusion imaging research including the Open Science Initiative for Perfusion Imaging (OSIPI) [47] this has potential for significant advancement.

Further prostate imaging biomarker studies are required to test and validate imaging biomarkers, which our team is conducting via multi-centre longitudinal clinical trials (ACTRN12618001810202 and ACTRN12621001118897p). These trials aim to identify imaging biomarkers which are accurate, repeatable and reproducible for early response assessment after prostate RT [48]. 

## Conclusions

DCE MRI parameters Ktrans and TTP are the most promising quantitative and semi-quantitative biomarkers respectively, for identifying prostate tumours at a voxel-level. Incorporating DCE MRI parameters with DWI and T2w in tumour classification models results in improved accuracy for tumour detection for both low and high grade tumours. DCE MRI parameters should therefore be used alongside DWI and T2w imaging to characterise spatial tumour biology for biologically targeted radiation therapy planning. Further work is required to standardise DCE MRI parameter acquisition, processing, and reporting for this purpose.

## Data Availability

The dataset analysed during the current study are available from the corresponding author on reasonable request.

## Abbreviations

DCE: Dynamic contrast enhanced
DWI: Diffusion weighted imaging
T2w: T2-weighted
PCa: Prostate cancer
ADC: Apparent Diffusion Coefficient
PI-RADS: Prostate Imaging and Reporting System
bpMRI: Biparametric MRI
mpMRI: Multiparametric MRI
ROI: Regions-of-interest
PSA: Prostate specific antigen
IRE: Initial rate of enhancement
IRW: Initial rate of washout
TTP: Time to peak enhancement
ME: Maximum enhancement
Tonset: Time of contrast agent onset
Twashout: Time of contrast agent washout
AUC: Area under the curve
Ktrans: Volume transfer constant
Ve: Volume of extra-vascular extra-cellular space
iAUGC60: Initial area under the Gadolinium contrast agent concentration curve for the first 60 s
AIF: Arterial input function
